# Human Papillomavirus-Associated Lymphoepithelioma-Like Carcinoma of the Anal Canal: A Case Report and Literature Review

**DOI:** 10.3389/fmed.2021.766960

**Published:** 2021-11-19

**Authors:** Weiwei Weng, Weiqi Sheng, Lei Wang

**Affiliations:** ^1^Department of Pathology, Fudan University Shanghai Cancer Center, Shanghai, China; ^2^Department of Oncology, Shanghai Medical College, Fudan University, Shanghai, China; ^3^Institute of Pathology, Fudan University, Shanghai, China

**Keywords:** human papillomavirus, lymphoepithelioma-like carcinoma, anal canal, immune microenvironment, case report

## Abstract

Lymphoepithelioma-like carcinoma is a rare type of tumor that is histologically identical to lymphoepithelial carcinoma of the nasopharynx. Lymphoepithelioma-like carcinomas (LELCs) are closely associated with viral infections. Human papillomavirus (HPV)-associated LELCs have been reported in a variety of anatomic sites. We reported an extremely rare case of a 25-year-old woman with LELC derived from the anal canal, which is the second case reported at this site. The tumor was diffusely positive for p16 staining, and was correlated with high-risk HPV-16; Epstein-Barr virus-encoded small RNA was negative; PD-L1 positivity and abundant CD8+ T cell infiltration were observed, indicating a “hot” immune microenvironment. In reporting this case, we highlight the potential for misdiagnosis and suggested an association of HPV infection with LELC in the anal canal.

## Introduction

Lymphoepithelioma refers to a syncytial growth pattern of undifferentiated malignant cells with prominent non-malignant lymphoplasmacytic stromal infiltration, which was originally described as a neoplasm of the nasopharynx. Tumors with morphology similar to that of nasopharyngeal lymphoepithelioma have been termed lymphoepithelioma-like carcinomas (LELCs). LELCs have been previously identified in several anatomic sites other than the nasopharynx, with a relatively low incidence, such as in the salivary gland (1), lung (2), thymus (3), stomach (4), urinary tract (5), uterine cervix (6), oral cavity (7), breast (8), and skin (9).

Lymphoepithelioma-like carcinomas have been reported to be closely related to viral infections. Epstein-Barr virus (EBV) infections have been purported to be involved in the etiology of lymphoepithelioma of the nasopharynx (10). It was previously described that EBV infection was also associated with most LELCs in foregut-derived organs, such as the salivary gland (11), thymus (12), stomach (4), intra-hepatic biliary epithelium (cholangiocarcinoma) (13), and lung (2), and, rarely but occasionally, in hindgut-derived organs, such as the colon (14). However, in some other so-called non-foregut organs, EBV infection may be less important. LELCs of the liver were reported to be associated with hepatitis B virus (HBV) and hepatitis C virus (HCV) infections (15, 16), while some LELCs derived from the gynecologic tract (17) were found to be human papillomavirus (HPV)-related.

Human papillomavirus-associated LELC is rarely derived from anal canal. To our knowledge, only one case of anal canal-derived LELC has been described previously, which is by Scott et al. (18). Herein, we report a new case of HPV-associated LELC originating from the anal canal with high PD-L1 expression and abundant CD8+ T cell infiltration.

## Case Description

A 25-year-old woman was admitted for intermittent hematochezia. A digital rectal examination revealed a polyp 20 mm above the pectinate line. A full clinical investigation revealed no tumors elsewhere. A liquid-based cytology test combined with a human HPV-DNA test was performed to exclude cervical disease. The patient underwent a transanal resection, and the sessile rectal polyp (25 mm) was removed.

**Figure 1:**
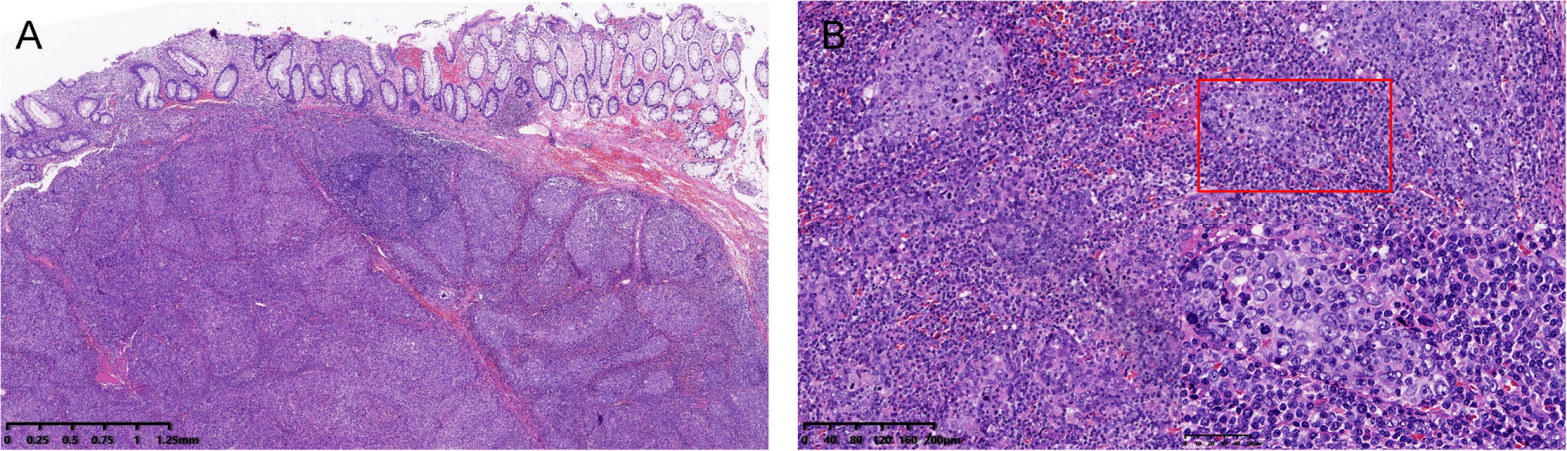
**(A)** Low-power view showing a submucosal tumor with relatively clear boundaries (hematoxylin and eosin, H&E, 2× magnification). **(B)** High-power view of the tumor showing a syncytial cytoplasmic appearance and abundant intratumoral immune infiltration (H&E. 10× and 40× magnification).

Histologically, a poorly differentiated submucosal tumor with relatively clear boundaries was discovered beneath the non-neoplastic rectal mucosa. The submucosal tumor showed a lymphoepithelioma-like growth pattern (Figure 1). The tumor was composed of sheets of large tumor cells with oval or round vesicular nuclei and prominent nucleoli, and had a syncytial cytoplasmic appearance. The intratumoral stroma was densely infiltrated with lymphocytes and plasma cells. The tumor invaded the submucosa to a depth of 11 mm, and the deep and radial margins were clear.

**Figure 2:**
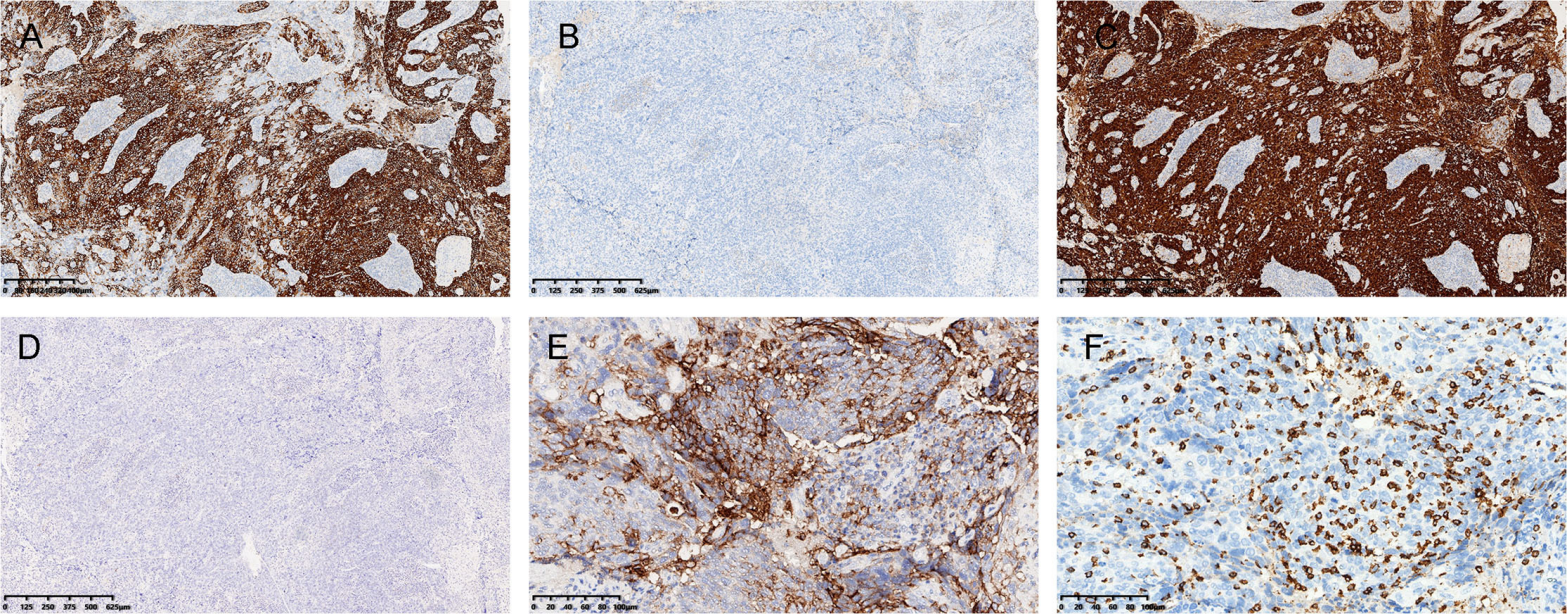
Immunohistochemistry and ISH. **(A)** The tumor was diffusely positive for CK5/6 **(A)**, and negative for CK20 **(B)**. **(C)** Immunostaining for p16 was diffusely positive, and **(D)** *in situ* hybridization (ISH) for Epstein-Barr virus-encoded small RNA was negative **(D)**. **(E)** Strong PD-L1 positivity and **(F)** abundant CD8+ T cell infiltration were observed. [**(A–D)**, 4× magnification; **(E,F)**, 20× magnification].

Immunohistochemically, the tumor cells were positive for pan-CK, P63, P40, and CK5/6 (Figure 2A), and negative for SATB2, Villin, CDX2, and CK20 (Figure 2B). Diffuse block-type immunoreactivity (diffuse nuclear and cytoplasmic staining) of p16 was shown (Figure 2C). Therefore, a diagnosis of poorly differentiated squamous carcinoma with lymphoepithelioma-like morphology was made.

Since LELCs have been previously reported to be closely related to EBV infection, *in situ* hybridization for EBV-encoded small RNA (EBER) was performed. The tumor cells were negative for EBER, while strongly positive staining was observed in the external positive control (Figure 2D). Linear array HPV genotyping (Yaneng Bio, Shenzhen, China) was performed for the detection of 23 HPV types, namely, 17 high-risk HPV types (16, 18, 31, 33, 35, 39, 45, 51–53, 56, 58, 59, 66, 68, 73, and 82) and 6 low-risk HPV types (6, 11, 42, 43, 81, and 83). High-risk HPV-16 was detected in this case. Therefore, this was a case of HPV-associated LELC of the anal canal.

Given that there was a strong local immune infiltration in the tumor microenvironment, immunostaining was performed for DNA mismatch repair (MMR) proteins, PD-L1, and CD8. The tumor showed intact nuclear staining of four MMR proteins (MLH1, PMS2, MSH2, and MSH6). Normal colonic mucosa (adjacent to the carcinoma) and lymphocytes served as positive internal controls. The immunostaining intensity of PD-L1 was evaluated by the combined positive score (CPS) (19), and a strong immunoreaction was observed with a CPS > 70 (Figure 2E). We also analyzed the presence of intratumoral CD8+ T cells and found that abundant tumor-infiltrating lymphocytes (TILs) were CD8-positive (Figure 2F).

The patient subsequently underwent further endoscopic evaluation and mapping biopsies. Neither endoscopic nor microscopic examination revealed any abnormalities. Thereafter, the patient received three cycles of chemotherapy (paclitaxel + Cisplatin + capecitabine) combined with concurrent radiotherapy. The patient continued to be asymptomatic with no signs of recurrence or metastasis 8 months post-operation.

## Discussion

Lymphoepithelioma-like carcinoma is an extremely rare type of tumor. Histologically, it mimics nasopharyngeal carcinoma. Viral infections may have an oncogenic role in the tumorigenesis of LELC. Several research groups have found a strong association between HPV and LELCs originating from so-called non-foregut sites, which are summarized in Table 1. Generally, the tumors tend to occur in late adulthood (range is from 26 to 93 years; median age is 57 years). The uterine cervix was the most frequently reported site. The penis, breast, vagina, rectum, and anal canal were also involved in a minority of cases. Regarding the subtype of HPV, high-risk HPV-16 and−18 were the most common types of HPV (detection rate: 63.4 and 14.6%, respectively), which were also the most common types of HPV associated with invasive squamous cell carcinomas (up to 41% for HPV-16 and 22% for HPV-18) (35, 36). It is reasonable to postulate that HPV may play a role in LELC that is similar to that in invasive squamous cell carcinomas.

**Table 1:** Literature review of HPV-related lymphoepithelioma-like carcinomas (LELCs).

**Reference**	**Site**	**Number of cases**	**Patients' age**	**HPV infection rate**	**Method**	**HPV subtype**
Tseng et al. (17)	Uterine Cervix	15	37–72	3/15	PCR	16
Tseng et al. (20)	Uterine Cervix	1	71	1/1	PCR	16
Noel et al. (21)	Uterine Cervix	2	53, 56	2/2	PCR	16, 18
Saylam et al. (22)	Uterine Cervix	1	72	1/1	PCR	18
Bais et al. (23)	Uterine Cervix	1	44	1/1	PCR	16, 45
Kulka et al. (24)	Breast	1	42	1/1	PCR	18, 33
Chao et al. (25)	Uterine Cervix	10	40–67	8/9	PCR	16, 18, 31, 35, 58
Nio et al. (26)	Breast	1	45	1/1	ISH and PCR	33
Kyozuka et al. (27)	Uterine Cervix	1	31	1/1	PCR	16, 71
Cañete-Portillo et al. (28)	Penis	12	54–92	12/12	DEIA	16, 33, 58, 66
Lloyd et al. (29)	Uterine Cervix	1	59	1/1	NA	HR
Koufopoulos et al. (30)	Breast	1	57	1/1	PCR and ISH	16
Pinto et al. (31)	Uterine Cervix	8	26–81	6/8	PCR and ISH	16, 18, 33
Scott et al. (18)	Vagina	2	53, 93	NA	NA	NA
Kim et al. (32)	Vagina	1	79	1/1	PANA RealTyper HPV Kit	16
Machado-Neveset al. (33)	Penis	1	50	NA	NA	NA
Kopparthy et al. (34)	Rectum	1	51	1/1	ISH	16

*NA, not available; HR, high risk; ISH, in situ hybridization; HPV, human papillomavirus; PCR, polymerase chain reaction*.

Human papillomavirus -associated LELC originating from the anal canal is extremely rare. To date, only two cases have been reported in English literature, including the present case (Table 2). The patients included one female and one male. The age of the patients was 68 and 25 at diagnosis, respectively. The male patient had no clinical symptoms and was diagnosed in a bowel cancer screening program, while the female patient was initially admitted because of rectal bleeding. The tumor sizes were relatively small, ranging from 8 to 25 mm. The presence of high-risk type HPV-16, not EBV, was observed in both cases. Both of the patients presented with localized neoplasms but with no evidence of metastasis. Follow-up of the present case revealed no evidence of recurrence after 8 months, whereas the prognostic data of the male patient were not available.

**Table 2:** Reported cases of HPV-related LELCs originating from the anal canal.

**Reference**	**Age**	**Sex**	**Symptom**	**Procedure**	**Size (mm)**	**EBV**	**HPV**	**p16 staining**	**Outcome**
Scott et al. (18)	68	Male	detected in a Screening Program	Transanal resection followed by chemoradiotherapy	8	negative	HPV-16	positive	NA
Present case	25	Female	rectal bleeding	Transanal resection followed by chemoradiotherapy	25	negative	HPV-16	positive	No sign of recurrence after 8 months

*NA, not available; HPV, human papillomavirus*.

Although LELC of the anal canal appears to be extremely rare, knowledge of this variant is critical to properly classify analogous tumors. The diagnosis of LELC depends on a comprehensive morphological examination along with immunohistochemical and molecular analyses. Tumors with a syncytial cytoplasmic appearance, as well as a dense stromal inflammatory infiltrate, are deemed to show a lymphoepithelioma-like growth pattern. Further immunostaining and molecular analyses would help prevent misdiagnosis, particularly of lymphoma, melanoma, neuroendocrine carcinoma, poorly differentiated carcinoma, medullary carcinoma, etc. In our case of anal canal LELC, diffuse positive cytoplasmic staining of pan-CK would help to prevent the misdiagnosis of lymphoma and melanoma. Neuroendocrine carcinoma constantly expresses neuroendocrine markers that are not normally expressed in LELCs. Abundant lymphocytic infiltration and the absence of gland formation prevent the tumor from being misdiagnosed as poorly differentiated adenocarcinoma. As for rectal medullary carcinoma, morphologic overlap does exist between this rare subtype of colorectal adenocarcinoma and LELC (37). The loss of expression of glandular epithelial cell markers, such as CDX2 and CK20; positive expression of squamous cell carcinoma markers, such as P40, P63, CK5/6; intact nuclear staining of MMR proteins and the HPV-related biomarker p16 help to determine the classification of the tumor type. In female patients, metastatic cervical cancer should also be taken into consideration. The differential diagnosis relies on comprehensive history-taking and gynecological examination. Accurate diagnosis and correct categorization should be made based on morphology, immunocytochemistry, and virology, as well as adequate history-taking. The characteristic morphology of syncytial growth pattern in the background consisted of prominent lymphoid stroma, and the propensity for tumor cells to be infected with HPV, warrant classification as HPV-related LELC.

There are no established treatment guidelines for LELC. Both patients with anal canal LELC were treated with transanal resection followed by concurrent chemoradiotherapy. Considering the rapid advancement and remarkable survival benefits for various tumor types, immunotherapy is now considered to be a promising cancer treatment, including for LELCs. It has been reported that a few unresectable, advanced LELCs originating from the liver and lung responded favorably to immune checkpoint inhibitors (38, 39). The expression of PD-L1 and cytotoxic T cells expressing cell-surface CD8 has emerged as a potential predictive biomarker for immunotherapy response (40, 41). Positive PD-L1 expression had been previously reported to be high in LELCs of the uterine cervix (31) and thymus (3). In the present case, we also found a relatively high PD-L1 expression level, with a CPS > 70. Abundant intratumoral CD8^+^ cytotic T-cells were also observed, suggesting a “hot” immune microenvironment. Therefore, we postulate that this patient could potentially benefit from immune checkpoint inhibitors, such as nivolumab or pembrolizumab.

Given its low incidence, the prognosis of LELC is poorly understood. Similar to what has been reported previously, patients with cervical or pulmonary LELCs have more favorable outcomes and significantly prolonged progression-free survival and overall survival times (17, 25, 31, 42). In LELC of the salivary glands, despite frequent locoregional lymph node and distant metastases, the 5-year survival rate is still high (43). LELCs tend to exhibit an indolent behavior (8). Complete resection may be curative for a particular type of LELC (44). Inflammatory infiltrates in the stroma reflect both the humoral and cell-mediated immune responses of the patients to the tumor, resulting in decreased lymph node metastases, suggesting a favorable prognostic indicator (6). The number of LELCs, especially those originating from the anal canal, is still limited; more studies with longer follow-up periods should be carried out to better define the clinical behavior of these tumors.

## Conclusion

In summary, we reported a rare case of LELC of the anal canal and reviewed the previous literature on HPV-related LELCs. The tumor was associated with high-riskHPV-16. Strong PD-L1expression and abundant CD8+ T cell infiltration suggested that the tumor may benefit from immunotherapy.

## Data Availability Statement

The original contributions presented in the study are included in the article/supplementary materials, further inquiries can be directed to the corresponding author/s.

## Ethics Statement

Written informed consent was obtained from the individual(s) for the publication of any potentially identifiable images or data included in this article.

## Author Contributions

WW analyzed and interpreted the patient data and was a major contributor to the writing of the manuscript. WS interpreted the pathological data. LW interpreted the clinical data. LW and WS substantively revised the manuscript. All authors contributed to the article and approved the submitted version.

## Conflict of Interest

The authors declare that the research was conducted in the absence of any commercial or financial relationships that could be construed as a potential conflict of interest.

## Publisher's Note

All claims expressed in this article are solely those of the authors and do not necessarily represent those of their affiliated organizations, or those of the publisher, the editors and the reviewers. Any product that may be evaluated in this article, or claim that may be made by its manufacturer, is not guaranteed or endorsed by the publisher.
